# The magnitude of Yo-Yo test improvements following an aerobic training intervention are associated with total genotype score

**DOI:** 10.1371/journal.pone.0207597

**Published:** 2018-11-28

**Authors:** C. Pickering, J. Kiely, B. Suraci, D. Collins

**Affiliations:** 1 Institute of Coaching and Performance, School of Sport & Wellbeing, University of Central Lancashire, Preston, United Kingdom; 2 Exercise and Nutritional Genomics Research Centre, DNAFit Ltd, London, United Kingdom; 3 Suraci Consultancy, Portsmouth, United Kingdom; Sao Paulo State University - UNESP, BRAZIL

## Abstract

Recent research has demonstrated that there is considerable inter-individual variation in the response to aerobic training, and that this variation is partially mediated by genetic factors. As such, we aimed to investigate if a genetic based algorithm successfully predicted the magnitude of improvements following eight-weeks of aerobic training in youth soccer players. A genetic test was utilised to examine five single nucleotide polymorphisms (*VEGF* rs2010963, *ADRB2* rs1042713 and rs1042714, *CRP* rs1205 & *PPARGC1A* rs8192678), whose occurrence is believed to impact aerobic training adaptations. 42 male soccer players (17.0 ± 1y, 176 ± 6 cm, 69 ± 9 kg) were tested and stratified into three different Total Genotype Score groups; “low”, “medium”and “high”, based on the possession of favourable polymorphisms. Subjects underwent two Yo-Yo tests separated by eight-weeks of sports-specific aerobic training. Overall, there were no significant differences between the genotype groups in pre-training Yo-Yo performance, but evident between-group response differentials emerged in post-training Yo-Yo test performance. Subjects in the “high” group saw much larger improvements (58%) than those in the ‘medium” (35%) and “low” (7%) groups. There were significant (p<0.05) differences between the groups in the magnitude of improvement, with athletes in the “high” and medium group having larger improvements than the “low” group (d = 2.59 “high” vs “low”; d = 1.32 “medium” vs “low”). In conclusion, the magnitude of improvements in aerobic fitness following a training intervention were associated with a genetic algorithm comprised of five single nucleotide polymorphisms. This information could lead to the development of more individualised aerobic training designs, targeting optimal fitness adaptations.

## Introduction

Aerobic capacity (as determined by maximal oxygen uptake, VO_2max_) is considered crucial for sports performance. The greater the aerobic capacity of an athlete, the longer they can exercise at a given intensity [1]. Additionally, aerobic fitness enhances recovery from high intensity intermittent exercise, such as that found in most team sports [2], and also potentially differentiates between performance levels, with elite team-sport athletes scoring higher than their sub-elite and amateur counterparts on tests of aerobic fitness [3,4]. Furthermore, improvements in aerobic fitness following training have been associated with improvements in soccer performance [5]. As such, aerobic fitness training is a fundamental inclusion in most professional team-sport physical preparation programmes.

Similarly, within endurance sport training there is on-going debate, in both the academic and coaching domains, focused on uncovering the “best” combination of running volumes and intensities necessary to optimally drive positive adaptation, and hence improve performance [6]. However, the belief that there is a universal “best” type of training to develop aerobic performance is predicated on the implicit assumption that athletes respond to the imposed training demands in a broadly similar fashion. In recent years, this conventional presumption has been challenged by empirical evidence showing unexpectedly extensive inter-individual in aerobic fitness gains experienced by participants undertaking identical training interventions [7–10]. This inter-individual response diversity is exemplified by the collection of studies constituting the HERITAGE (HEalth, RIsk factors, exercise Training And GEnetics) Family Study; whilst the mean improvement in aerobic fitness following training was 19%, some subjects saw improvements as high as 40%, whilst others experienced no improvements [8]. Further analysis of the HERITAGE data revealed that genetic variation between subjects explained approximately 47% of this variance [8], although such data has recently been critically evaluated [11]. Such extensive inter-individual variability has been replicated in a number of other studies examining adaptations to aerobic training [7,9,10].

The demonstrated magnitude of inter-individual adaptive response following aerobic training poses a potential problem to conventional exercise prescription methodologies. For example, professional athletes may fail to elicit expected benefits, and patients prescribed aerobic exercise–under the premise that such training will improve health parameters–may fail to realise meaningful benefits, despite engaging in the recommended training. Since the completion of the HERITAGE Family Study, the field of sports genetics has grown exponentially. Currently, 155 genetic markers are associated with elite athlete status [12], and more still are associated with training response [13]. However, the translation and application of this research to both sports training and general health contexts remains both tentative and controversial [14].

Previously, research has focused on exploring the influence of genetic variations on elite endurance athlete status, with a general lack of predictive ability of these variations [15,16]. However, with heritable factors potentially accounting for close to half of the variation in exercise response between individuals [8], there is the potential that insight into the genetic profile of the individual could improve exercise programme design. Research on the impact of genetic variation on exercise adaptation has identified a series of single nucleotide polymorphisms (SNPs) which may contribute to observed differences in response to aerobic training. Five of these SNPs from four different genes (*VEGF* [17], *PPARGC1A* [18], *CRP* [19,20], and two from *ADRB2* [21–23]) have been collated into an algorithm used in a commercially available test. These SNPs affect different dimensions of cardiovascular function, and are associated with either VO_2max_ scores, or improvements in this capacity following aerobic training.

Given the observable inter-subject variations in training-induced aerobic adaptations, the ability to identify individuals who may exhibit smaller fitness gains could enable the evolution of more personalised training programme designs. Such an innovation would promote greater overall improvements within populations, enhancing training efficiency and increasing the chances of positive adaptation in a greater number of individuals. Therefore, the purpose of this study was to determine whether a genetic algorithm was associated with the magnitude of improvements in aerobic fitness in a group of youth soccer players following an eight-week training block. It is believed that players with a greater number of positive alleles for genes associated with higher aerobic fitness would see larger improvements following aerobic training than those with fewer positive alleles. A secondary aim is to attempt to bridge the gap between genetics research and sports science practice. The ability to utilize genotype assessment panels to inform training programme design holds the potential to revolutionise exercise prescription in medical, health and sporting domains. Yet genetic research, whilst potentially impactful, can often appear confusing to field-based practitioners and athletes, who require real-world data to inform their decision-making processes [24]. As such, this work is framed as a training observation study, as opposed to a genetic association study. The outcomes may provide meaningful, actionable training insights promoting the strategic incorporation of genetic information into training programme designs.

## Methods

### Subjects

Following University of Central Lancashire Ethics Committee approval according to the Declaration of Helsinki, a convenience sample of 42 male soccer players aged between 16–19 years of age (height 176 ± 6 cm, body mass 69 ± 9 kg) from a college soccer academy volunteered to participate in this study. Such a sample was chosen to best represent the size of a typical soccer squad. Each player had an average of 11 years’ football training experience, and was actively competing in the English College Football Association Leagues. All players aged 18 or over signed an informed consent form, with players aged under-18 co-signing the informed consent form along with their legal guardians.

### Methodology

Subjects were in a phase of training aimed at increasing aerobic capacity via sport specific conditioning, in this case small sided games. Sessions took place twice a week for the eight-week training block. Within each session, the subjects participated in small-sided games on pitches of differing sizes and with a different number of players, ranging from 3 v 3 to 5 v 5. The work periods were uniform in all sessions, consisting of four sets of four-minutes exercise and three-minutes of active recovery. All sessions were supervised by a UEFA A Licensed coach, who set and monitored the intensity of each training session, through the use of Rating of Perceived Exertion (RPE). The subjects also played in a minimum of one competitive match per week during this time, as the training intervention took place during the competitive season, specifically January to March. No additional physical training was prescribed during the intervention period. Subjects did take part in their normal technical and tactical training, which had a target RPE score of below 6. There was no control group, as requesting a group of competitive footballers to refrain from exercise is potentially in violation of the Declaration of Helsinki, and is almost certainly unethical [25].

Before and after the training block, subjects’ aerobic fitness was assessed by the Yo-Yo Intermittent Recovery Test, level 1 (Yo-Yo IR1), a reliable and valid measure of aerobic fitness [26]. Briefly, the test is comprised of repeated 2 x 20 m runs back-and-forth performed to an audible beep, separated by an active rest period of 10 seconds. The time allowed for each 20 m section decreases as the test progresses, resulting in a faster required running speed; this begins at 10 km·h^-1^, and is increased by 2 and then 1 km·h^-1^ for the respective next two speed levels. After this, the speed increases by 0.5 km·h^-1^ for each additional level. The test is halted when a subject fails to cover the distance in the required time on two consecutive occasions, indicating that exhaustion has occurred. All subjects were provided with verbal encouragement during the test. Subjects refrained from caffeine for at least 12 hours, and training for at least 24 hours, prior to testing, which took place outdoors on a soccer pitch, at the same time of day on both occasions. Individual results were expressed as distance covered in metres. Subjects had carried out Yo-Yo tests previously, and were fully accustomed to the assessment protocol.

### Genetic testing

Alongside the training programme, subjects underwent genetic testing using a commercially available self-testing kit from DNAFit Ltd. Subjects provided a saliva sample, collected using a sterile buccal swab. The samples were sent to IDna Genetics Laboratory (Norwich, UK), where DNA was extracted and purified using the Isohelix Buccalyse DNA extraction kit BEK-50 (Kent, UK), and amplified through PCR on an ABI7900 real-time thermocycler (Applied Biosystem, Waltham, USA). Through this process, genetic information regarding SNPs determined to affect aerobic trainability (*VEGF* rs2010963, *ADRB2* rs1042713 and rs1042714, *CRP* rs1205 & *PPARGC1A* rs8192678) [17–23] was determined. Each allele was given a score of between 0 and 4 points depending on the expected magnitude of its impact on improvements in aerobic fitness with training. The strength of the rating was based on the evidence from cumulative literature results averaged over time. The sum of these points was combined to give an overall score. This method is identical to Jones et al. [27], and similar to the methods used in other studies utilising genetic algorithms [28,29]. The subjects were stratified into three groups; “low”, “medium” and “high” depending on their weighted total genotype score (TGS), with a higher score indicating possession of a greater amount of alleles expected to improve adaptation to aerobic training. Those with an overall score of 40% or less were classed as “low”. Scores of 41–70% were classed as “medium”. A score of >70% was classed as “high”. These divisions were used in the absence of previous work, and represents a gross sub-division into categories based on the expectation that approximately 60% of subjects have a score of between 40–70% [30]. The athletes were blinded to their genetic results until completion of the final testing.

### Statistical analysis

Means, standard deviations and 90% confidence intervals (CI) were calculated for whole group and sub-groups for both pre- and post-training test scores. 90% CI were used as per the recommendations of Sterne and Smith [31] and Hopkins et al. [32]. These were examined by a 3 X 2 (Group X Time) mixed methods ANOVA, with repeated measures on the second factor. The dependent variable was the Yo-Yo scores (pre- and post-) obtained by each participant. Tukey’s HSD was also run. To further discover the differences between groups, pre- and post-training test scores were compared within groups using a paired sample t-test, and between groups using unpaired t-test. Statistical significance was set as P ≤0.05, which after adjustment using Bonferroni correction led to a significance level of 0.008 for the six t-tests. Cohen’s d was calculated for within- and between-group effect size. The thresholds used were <0.2 (trivial), 0.21–0.5 (small), 0.51–0.8 (moderate), 0.81–1.2 (large), 1.21–2 (very large), >2 (huge) [33,34]. Data were analysed using Microsoft Excel 15.29 (Microsoft Corporation, Redmond, WA, USA) and IBM SPSS Statistics 23 (IBM Corporation, Armonk, NY, USA).

## Results

There were no significant differences between the three different genotype groups at baseline in terms of age (low 17.2 ± 0.8y; medium 17.2 ± 1.0y; high 16.8 ± 1.1y), height (low 173.8 ± 3.9 cm; medium 177.4 ± 7.4 cm; high 174.9 ± 4.1 cm) or body mass (low 63.8 ± 9.3 kg; medium 70.0 ± 8.9 kg; high 71.2 ± 9.4 kg).

Table 1 illustrates the genotype-group data. After examination with a 3 X 2 (Group X Time) mixed methods ANOVA, there was a significant main effect of time (F (1, 39) = 67.8, P <0.001) and a significant interaction (F (2, 39) = 10.9, P <0.001). The main effect of Group (F (1,39) = 5.11) was not significant.

**Table 1:** Pre- and post-training Yo-Yo test scores, stratified for individual genotype groups.

Group	Pre-training Yo-Yo Score (m) [mean (SD; 90% CI)]	Post-training Yo-Yo Score (m) [mean (SD; 90% CI)]	P-Value (paired t-test)	Effect Size (Cohen’s d) (90% CI)
Low (n = 6)	1006 (292; 766 to 1247)	1073 (281; 842 to 1304)	0.0041	0.23 “Small”
Medium (n = 23)	1045 (472; 876 to 1213)	1409 (453; 1246 to 1571)	<0.0001	0.79 “Moderate”
High (n = 13)	969 (493; 725 to 1212)	1529 (508; 1278 to 1780)	<0.0001	1.12 “Large”

The significant main effects of Time supports the impact of the aerobic training intervention, as all groups showed an improvement in fitness. In contrast, follow up on the between group main effect using Tukey’s HSD showed no significant differences (all pairwise comparisons non-significant). As such, groups were taken as being equivalently fit at baseline.

The interaction effects were of most interest, in that these addressed the main purpose of the study. Building on the significant overall differences demonstrated by the significant interaction, follow up was conducted by use of three paired t-tests on the before and after data of the three groups. These results are shown in Table 1.

**Fig 1:**
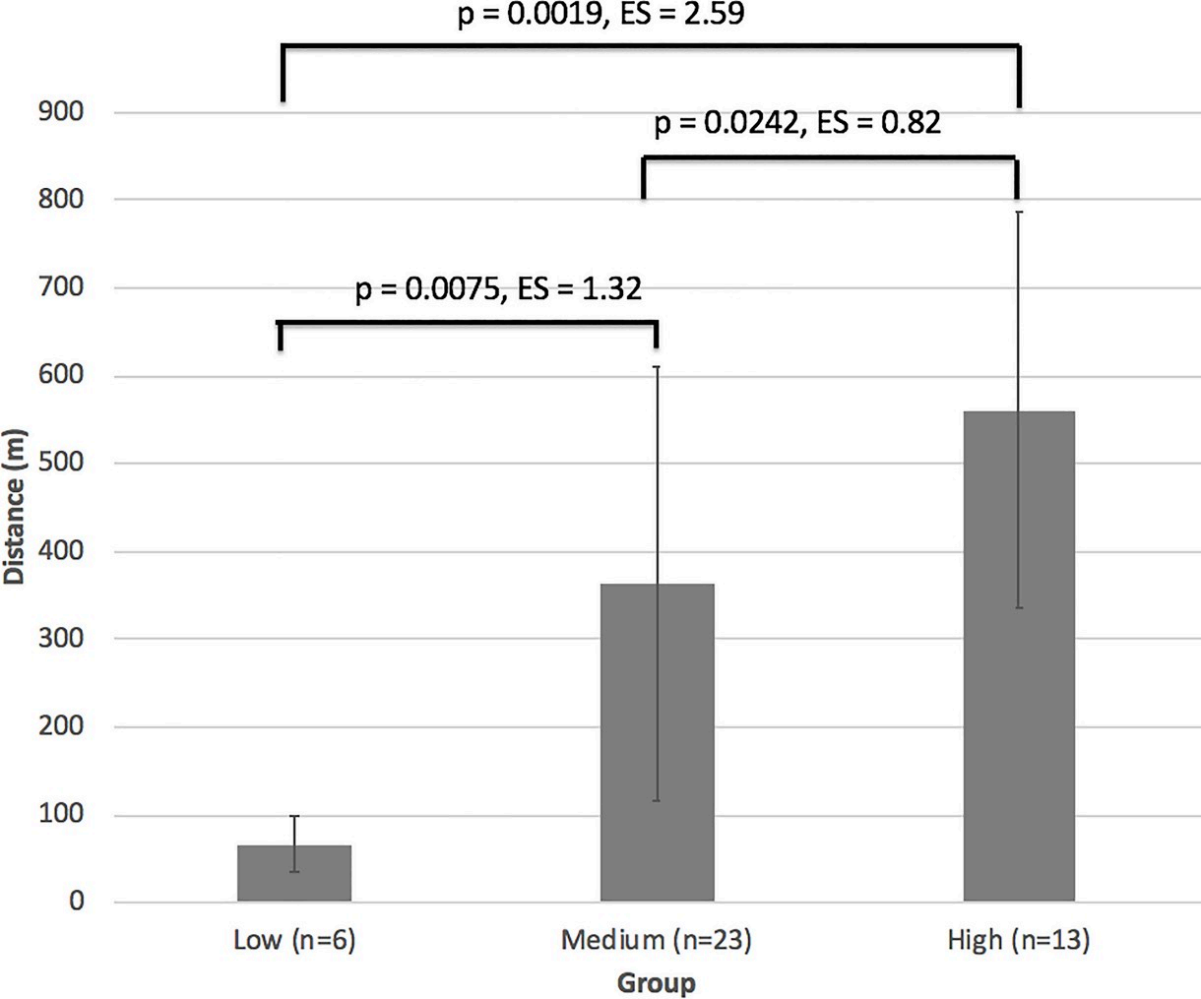
Between group interactions for post-training improvements in Yo-Yo score.

We then analysed the data for between-group interactions, which is summarised in Fig 1. The key finding is that there was a significant difference (P < 0.05) between all groups, which remained after Bonferroni correction for differences between “low” and “high”, and “low” and “medium” comparisons. The effect sizes were very large (1.32 for the difference between “low” and “medium” groups, large (0.82 for differences between “medium” and “high”, and huge (2.59 for differences between “low” and “high” groups.

**Fig 2:**
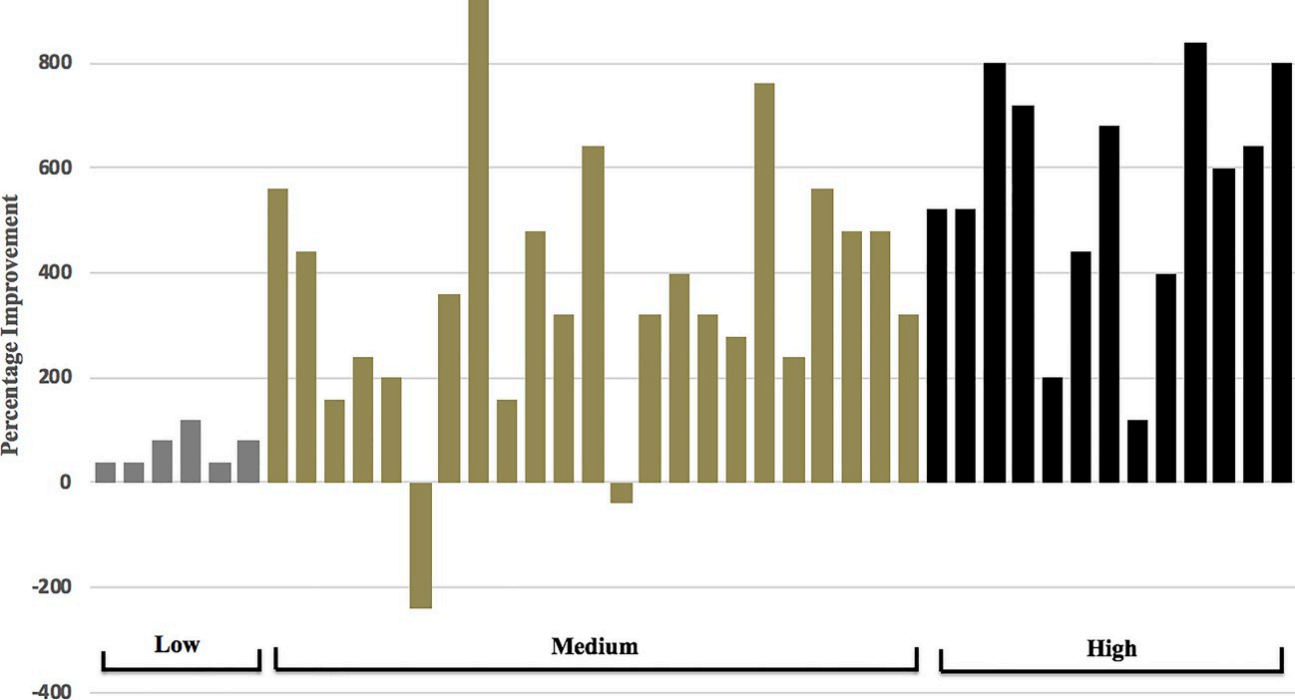
Individual percentage improvement scores across “low”, “medium” and “high” groups.

In all groups, the mean improvement was 382 ± 270 m (90% CI 312 to 452 m), which represents an improvement of 37.5%. Within the “low” group, the mean improvement was 67 ± 33 m (90% CI 40 to 94 m), representing a mean improvement of 7.5%. No subject in the “low” group had an improvement greater than 120 m. In the “medium” group, the mean improvement was 364 ± 248 m (90% CI 274 to 452 m), representing a mean improvement of 43.8%. Within this group, two subjects exhibited a negative improvement (i.e. got worse), whilst all other subjects (21/23; 91%) showed improvements greater than 120 m. Five subjects (22%) from the “medium” group showed an improvement of greater than 500 m. In the “high” group, the mean improvement was 560 ± 225 m (90% CI 449 to 671 m), representing a mean percentage improvement of 72.6%. In the “high” group, 9/13 (69%) of subjects had an improvement of greater than 500 m, with all subjects (100%) showing an improvement of 120 m or greater. There was considerable inter-individual variation in magnitude of aerobic improvements between subjects, as illustrated in Fig 2.

## Discussion

The results of this study indicate that, following an eight-week training period, the magnitude of improvements in Yo-Yo test scores show significant inter-subject variation. This finding is in agreement with previous research examining variability in aerobic fitness improvements following training [8,35]. Crucially, the magnitude of training improvements was associated with a five SNP TGS determined by genetic profiling before training began.

The use of this genetic algorithm did not predict absolute performance in the Yo-Yo test. This observation adds to previous work suggesting that genetic testing should not be used as a talent identification tool [14]. However, the results of the algorithm were associated with the magnitude of improvements in Yo-Yo score following training. To illustrate how this algorithm does not predict aerobic “talent”, the lowest pre-training (440 m) and post-training (640 m) score occurred within a subject from the “high” genotype group. If genetic tests were to have utility in the prediction of talent, it would be expected that the lowest aerobic test scores would occur in the “low” group. However, this same subject’s test improvement (200 m) was greater than every subject in the “low” group. This supports the assertion that the genetic-based algorithm has utility in predicting training response, not talent. Similarly, when the two subjects who exhibited a reduction in Yo-Yo score in the post-training test are removed, every subject from the “medium” and “high” group showed improvements equal to (n = 1) or greater than (n = 25) those in the “low” group. Of the two subjects exhibiting lower post-training scores, one had a score reduction of 40m (from 2440 m to 2400 m), a 1.64% reduction, which is within the range of test-retest variation previously reported [26]. The second subject had a performance decrement of 240 m; which, whilst substantial, remains unexplained.

The potential to predict response to aerobic training may be useful to ensure that appropriately individualised training methods are utilised to maximise training adaptations. For example, if an individual is classed as having a low aerobic trainability, it might be prudent for them to follow a different training programme to an individual classed as having a high aerobic trainability. There are many ways to increase performance in aerobic endurance activities, including improvements in VO_2max_, running economy, lactate threshold, and VO_2_ kinetics [36]. In individuals with a low aerobic trainability, diverting training resources towards optimising improvements outside of VO_2max_ might be appropriate; there are various methods of achieving this, including resistance and plyometric training [37]. Knowledge of predicted training responsiveness can also lead to more personalised manipulation of common training factors such as volume, intensity, frequency and duration to improve exercise adaptation. As an example, it has been previously found that the number of low responders to an aerobic training intervention could be significantly reduced, and even eliminated, with an increase in exercise intensity [9]. Similarly, a recent paper found that an increase in exercise frequency and volume, with the same intensity, completely reduced the occurrence of non-response to aerobic training [38]. The demonstrated predictive validity of this genetic algorithm potentially adds useful information to coaches, aiding in the interpretation of fitness assessments, and ensuring information is available for the planning of more effective training programmes.

The SNPs utilised in this study occur within genes shown to affect either aerobic capacity, or the magnitude of improvements in aerobic fitness following exercise. Most of these SNPs occur in genes that affect the cardiopulmonary system or mitochondrial biogenesis. *VEGF* encodes for vascular endothelial growth factor, which impacts the growth of new blood vessels in and around skeletal muscle. The C allele of this common polymorphism (rs2010963) increases expression of this gene, likely leading to increased blood vessel growth and hence greater oxygen availability during exercise [17]. *ADRB2*, which has two common polymorphisms (rs1042713 and rs1042714) included in this algorithm, encodes for the β_2_-adrenergic receptor. This receptor is the site to which catecholamines can bind, increasing cardiovascular parameters such as stroke volume and cardiac output. These two common polymorphisms are associated with increases in receptor density, leading to increased stroke volume, cardiac output, vasodilation, and bronchodilation, all of which increase oxygen delivery. These polymorphisms may also increase exercise-based lipolysis, improving performance at lower exercise intensities [23], and have previous been associated with elite athlete status [22], and maximal oxygen consumption [21]. The *CRP* rs1205 polymorphism can lead to an increase in C-reactive protein release at both rest and during exercise, potentially negatively impacting VO_2max_ [19]. *PPARGC1A* encodes for PGC-1α, the master regulator of mitochondrial biogenesis. G allele carriers at rs8192678 typically have higher VO_2max_ values following exercise [18]. The SNPs used in this algorithm are not exhaustive, but represent those that have been well replicated. As other SNPs which impact improvements in aerobic fitness are discovered and replicated in multiple cohorts, their addition to this genetic algorithm would improve its association with aerobic fitness improvements.

Previous research exploring the genetic underpinning of soccer performance has explored the prevalence and impact of *ACTN3* and *ACE* within cohorts of Brazilian soccer players [39–41]. Whilst this was primarily explored with regard to soccer athlete status, subjects with the XX genotype of *ACTN3* were found to perform significantly better in an aerobic test compared to those with the RR genotype [39]. This finding, however, was not replicated by a later study [40], thereby demonstrating the importance of replication within exercise genomics studies, especially given the often small effect sizes of each individual SNP.

Regarding the practical application of these findings, astute coaches have long been aware that improvements in aerobic fitness following training vary extensively between athletes. This is true even when those athletes have similar training histories, dietary habits and lifestyles. In addition, prediction of adaptation to aerobic training is currently not possible using conventional physiological assessment tools [35]. This study suggests that a simple, non-invasive genetic test is associated with the magnitude of improvements in aerobic fitness following a training programme, and so may potentially help in the programming of training. The identification of athletes who are more likely to see smaller improvements allows for such athletes to follow a different training intervention, potentially with greater intensity (and therefore shorter exercise durations) [9], frequency [38] or perhaps with an increased emphasis on repeated sprint or resistance training. This contrasts with the current best practice, which is the application of training to an athlete, and the measuring of that response. If the response is less than expected, then either the athlete is considered to have reached their potential, or a different training method is utilised. This trial and error approach is costly in terms of time. Given that a high-level sporting career can last around 10 years, a training cycle spent doing ineffective training can seriously harm the athlete’s performance. The ability to more accurately predict the magnitude of exercise response could potentially:

1. Improve training prescription accuracy, and therefore training efficiency
2. Enhance the personalisation of athlete-specific training programmes
3. Reduce the costly trial and error process of executing unnecessary and/or inefficient training modalities.

These results potentially represent an early step on the journey to a higher level of personalisation within the training process. A possible limitation of this initial study is the modest sample size (n = 42). Nevertheless, whilst modest, this sample size compares well to similar research in this field [42–44]. This sample size is also representative of the size of a typical soccer squad (first and reserve teams), giving it real-world validity. The subjects were all male, so it is not clear if the results would be applicable for females. In addition, the number of subjects in the “low” group was small (n = 6); pre-test power calculations were not possible because the genetic results of the athletes were not available until completion of the study. With information regarding frequency of athletes expected to be in the “low” group now available, this information can be used to ensure adequate sample sizes in future. Further research should build on these initial findings in a larger cohort, other sports, and females, as well as studying interventions aimed at enhancing aerobic training response. The Yo-Yo IR1 test used in this study is a maximal test, and so scores are potentially influenced by subject motivation. Whilst none of the SNPs used in this study have been found to impact motivation, there is a small possibility that variation in these genes could influence exercise tolerance, and hence test performance [45]. Additionally, improvements in Yo-Yo test performance may occur outside of adaptations in aerobic capacity, such as improvements in technical performance and anaerobic capacity. Future studies may wish to use laboratory based tests to directly explore aerobic fitness improvements; however, in the present case, we wished to utilise a field-based test to ensure real-world validity. Additionally, as no comparator arm was present, there is the potential that random-within subject variation contributed to the observed inter-individual variation [46]. Furthermore, developmental factors, such as age (both chronological and developmental) may also have confounded these results. However, we found no significant different between the groups at baseline in terms of age, height, and body mass, suggesting that the groups were fairly matched in this regard. Finally, whilst the results of this study indicate that the current five-SNP algorithm has utility, the addition of more polymorphisms will enable it to become even more precise. Indeed, it is envisioned that the current algorithm is not a definitive end-point, but instead an initial attempt to predict training response that will become more refined and precise as more information is available. Nevertheless, the fact remains that very little research has been done in utilising genetic information in sporting practice, despite there being an undoubted genetic influence on the magnitude of adaptation following aerobic training. The novel findings of this study, even at this early stage in the evolution of such technology, should contribute to the further development of this area.

## Conclusions

The results of this study indicate there is considerable inter-subject variability in response to aerobic training in a group of well-trained male soccer players. In addition, we have also shown that a genetic test comprised of five SNPs is associated with the magnitude of these improvements. This previously unavailable information has the potential to provide insight to coaches, medical practitioners, personal trainers and athletes, enabling more informed decision making and evidence-led customisation of training programmes aimed at improving aerobic fitness. This potentially aids athletes, and their support staff, in selecting the optimal training modality, allowing for a more personalised training approach, and, in future, the maximisation of training adaptations for all athletes.

## Supporting information

S1 File Individual player data. (XLSX)
